# Harnessing machine learning for metagenomic data analysis: trends and applications

**DOI:** 10.1128/msystems.01642-24

**Published:** 2025-10-07

**Authors:** Shradha Sharma, Hari Priya Narahari, Karthik Raman

**Affiliations:** 1 Bhupat and Jyoti Mehta School of Biosciences, Indian Institute of Technology (IIT) Madras 37268, Chennai, Tamil Nadu, India; 2 The Centre for Integrative Biology and Systems medicinE (IBSE), Wadhwani School of Data Science and AI, IIT Madras 37268, Chennai, Tamil Nadu, India; 3 Department of Data Science and AI, Wadhwani School of Data Science and AI, IIT Madras 37268, Chennai, Tamil Nadu, India; 4 Department of Computer Science and Engineering, Shiv Nadar University, Chennai, Tamil Nadu, India; Florida Atlantic University, Boca Raton, Florida, USA

**Keywords:** metagenomics, microbiome, machine learning, deep learning

## Abstract

Metagenomic sequencing has revolutionized our understanding of microbial ecosystems by enabling high-resolution profiling of microbes across diverse environments. However, the resulting data are high-dimensional, sparse, and noisy, posing challenges for downstream data analysis. Machine learning (ML) has provided an arsenal of tools to extract meaningful insights from such large and complex data sets. This review surveys the existing state of ML applications in metagenomic data analysis, from traditional supervised and unsupervised learning to time-series modeling, transfer learning, and newer directions such as causal ML and generative models. We highlight certain key challenges and delve into important issues like model interpretability, emphasizing the importance of explainable AI (XAI). We also compare ML with mechanistic models, commenting on their relative advantages, disadvantages, and prospects for synergy. Finally, we preview future directions, such as the incorporation of multi-omics data, synthetic data generation, and Agentic AI systems, highlighting the increasingly prominent role that AI and ML will play in the future of microbiome science.

## INTRODUCTION

With the advent of high-throughput sequencing and computational innovations, our knowledge of microbial diversity across ecosystems has expanded tremendously. These advances have revealed previously unrecognized species, metabolic pathways, and ecological interactions that shape microbial communities. The structure and composition of these communities are determined by their environment, and interactions among microbes create system-level properties that, in turn, influence their environment. Therefore, the knowledge of these communities is fundamental to the understanding of biogeochemical cycling, host health, and soil nutrient dynamics. Nevertheless, the complexity and vastness of biological data pose computational challenges in processing and interpreting the data correctly. One of the significant challenges with metagenomic data is its inherent compositional nature. Since the overall read count (i.e., the total number of sequencing reads assigned to taxa) is limited by sequencing depth, the abundance of a single taxon is affected by the presence and abundance of others, creating spurious correlations and biases (1). This compositional nature of the data needs to be addressed through proper normalization and data transformation techniques. In addition, microbiome data are large, high-dimensional, highly variable, and plagued by the ”curse of dimensionality,” where the number of features (usually the abundances of different organisms) is much larger than the number of samples (2). This high dimensionality complicates data interpretation. In addition, microbiome data are often noisy and redundant, complicating downstream analysis and the discovery of meaningful biological patterns (2). To address these challenges, researchers are increasingly turning to ML techniques for more effective analysis and interpretation in microbiome studies.

While previous reviews on ML in microbiome have discussed in detail the data transformation and normalization techniques (3–5), the scope of this review is to provide an overview of ML approaches and applications for microbiome analysis, outlining their core concepts, strengths, and limitations. Our goal is to help researchers choose methods that best fit their data and objectives. We then describe the next steps, *post hoc* interpretability techniques, and quantitative scoring metrics to increase confidence in model results. Finally, we emphasize that ML is just one of many tools for metagenomic data analysis and that methodological choices should always be guided by the data at hand and the specific research question.

## CURRENT LANDSCAPE OF ML IN MICROBIOME RESEARCH

ML has become a key tool in microbiome research because it can handle complex, high-dimensional data and uncover patterns that traditional methods often miss. This is especially important given the noisy, sparse, and imbalanced nature of metagenomic data (2). In these data sets, features—that is, the relative abundances of different taxa are unevenly distributed, with many rare taxa and only a few dominant ones. This makes it difficult for models to generalize across samples. Feature engineering helps address this by transforming raw data to better capture the biologically relevant signals. Techniques broadly fall into two categories: feature extraction, which converts raw data into more structured and informative representations, and feature selection, which removes redundancy by retaining only the most relevant features. Several methods exist for each, but manually tuning the right combination of feature engineering and ML models is labor-intensive and error-prone. Automated approaches like BioAutoML (6) streamline this process by testing multiple pipelines to identify the most effective combinations, improving performance while reducing human effort.

Although robust feature engineering lays the foundation, the success of ML models also depends on how the learning process is structured, particularly in relation to data labeling. Currently, most of the ML models for microbiome analysis are based on supervised learning. This reflects the field’s fundamental need to answer concrete questions like pathogen detection, functional annotation, and disease prediction, which require labeled data sets. However, high-quality labeled data remain scarce for many important scenarios, including studies of rare diseases, specialized populations, or longitudinal studies. This limitation has driven interest in semi-supervised approaches like meta model agnostic pseudo label learning (MMAPLE), which employs a teacher–student framework to progressively improve predictions by leveraging both labeled and unlabeled data, even when dealing with out-of-distribution samples (7).

For cases where even semi-supervised learning struggles due to extreme data scarcity, transfer learning offers a powerful alternative by adapting models pre-trained on large data sets to smaller related ones. A prime example is EXPERT (8), which was first trained on the comprehensive MGnify (9) database before being fine-tuned for specific applications ranging from age-related microbiome changes to different stages of colorectal cancer.

As these diverse ML approaches continue to proliferate, the field faces growing needs for standardization and benchmarking to enable fair comparisons between methods. Critical assessment of massive data analysis (CAMDA) provides an open framework for metagenomic interpretation through community challenges (10), offering pre-curated data sets and defined metrics to objectively assess model performance. Similarly, critical assessment of metagenome interpretation (CAMI) benchmarks tools for assembly, taxonomic profiling, and binning using realistic data sets to ensure reproducibility (11).

With these developments in mind, it is essential to recognize the broad range of ML models currently used in microbiome research. Table 1 summarizes the recently developed ML tools and their applications in microbiome analysis. These tools span several ML methods, such as time-series analysis, supervised and unsupervised learning, and deep learning (DL) models, specifically created to address the specific challenges of microbiome data analysis.

**TABLE 1:** Cross-consistency matrix (CCM) of ML approaches and applications in metagenomic data analysis^*a*^

Major application category	Subcategory	Time-series		Supervised learning		Unsupervised learning	
		ML	DL	ML	DL	ML	DL
Assembly and binning	Assembly			MetaVelvet-SL (12)	MetaVelvet-DL (13)	Reinforcement learning (14)	VAMB (15)
	Binning			BusyBee (16)	SemiBin (17)	MaxBin (18), MaxBin 2.0 (19)	COMEBin (20)
	Bin refinement and quality check			CheckM2 (21)		Agglomerative hierarchical clustering (22)	SemiBin (17)
Annotation	Taxonomy			R-SVM (23)	CHEER (24)	Latent Dirichlet allocation (25)	DBN (26)
	Phylogenetic tree				DeePhy (27)	Split-weight embedding (28)	DeLUCS (29)
	Functional profile			Random forest (30)	Meta-MFDL (31)	PICA (32)	DeepARG (33)
Microbiome analysis	Prediction	MDITRE (34)	phyLoSTM (35)	KernelBiome (36)	MDL4Microbiome (37)	VALENCIA (38)	DeepGum (39)
	Host–microbe interaction		Recurrent neural network (40)	Random forest (41)	Meta-Spec (42)	RCCA (43)	MEGMA (44)
	Microbe–microbe interaction	mbtransfer (45)	MicroGrowthPredictor (46)	SVM (47)	LSTM framework (48)	Deep latent space (49)	Transformer (50)
	Pattern recognition	ARGfore (51)	DeepVirFinder (52), EXPERT (8)	Recursive ensemble feature selection (53)	BPNNHMDA (54)	DMM (55)	DeepBioGen (56)
Preprocessing	Feature engineering		DeepIDA-GRU (57)	BioAutoML (6)	MDeep (58)	Hierarchical feature engineering (59)	DeepGeni (60)
	Dimensionality reduction	EMBED (61)	TCAM (62)	Recursive feature elimination (63)	Autoencoder Neural Network (64)	Barnes-Hut stochastic neighbor embedding (65)	DeepMicro (66)
Synthetic data generation			DeepMicroGen (67)		DeepBioGen (56)		Evo 2 (68)

^*a*^ This matrix summarizes ML methods applied to various metagenomic analysis tasks. Columns represent ML paradigms, such as supervised, unsupervised, DL, and time-series approaches, while rows correspond to key application areas, including assembly, binning, taxonomic annotation, functional profiling, prediction of host-microbe interaction, and pattern recognition. Each filled cell indicates the existence of one or more tools that implement the corresponding ML method for the given task. Empty cells may be interpreted as (i) potential research gaps, (ii) infeasible method-task pairings due to data or modeling constraints, or (iii) areas outside the scope of this review.

## INTERPRETABILITY

ML and DL algorithms have demonstrated remarkable success in analyzing complex, high-dimensional data sets. However, despite their predictive power, the internal decision-making processes of these models often remain opaque, which is why they are characterized as “black-box” models. This lack of transparency arises from the inherent complexity and non-linearity associated with the information they analyze, making it difficult for investigators to trace how inputs determine outputs. In biological scenarios, where interpretability is key, dependency on model predictions without understanding their underlying rationale can be misleading or even detrimental. To overcome this, the emerging field of explainable AI (XAI) offers frameworks designed to illuminate model reasoning.

XAI primarily aims to deliver (i) interpretability, yielding qualitative insight into predictions, usually presented visually or as text; (ii) explainability, facilitating humans’ ability to understand the internal workings of a model; and (iii) causality, determining the degree to which a model replicates the underlying causal relations among inputs and outputs (69). Among the most widely used XAI techniques are *post hoc* explanation methods like LIME (70) and SHAP (SHapley Additive exPlanations) (71), which diagnose model behavior after training.

LIME was among the first general-purpose model-agnostic methods created to explain ML models. Its core idea is to approximate the model locally using a simpler, interpretable surrogate model. More precisely, LIME slightly perturbs the input data and looks at how the predictions change. It then uses a simple model, for example, linear regression, to fit these locally produced data, effectively pointing out which features (e.g., microbial taxa) had the largest influence on a given prediction. However, this local fidelity can also be a weakness since it might not capture the model’s global decision-making rationale. LIME is valuable for questions where sample-specific predictions are needed. For example, why did a model classify a microbiome sample as “high risk,” and which characteristics contributed most to that result? Nonetheless, owing to its surrogate nature, LIME’s explanations may not always align with the original model’s reasoning (72).

Another *post hoc* method, SHAP, has been of special interest due to its strong theoretical foundations and reproducible outputs. It applies Shapley values—derived from cooperative game theory—to attribute credit or blame to features based on how they contribute to a model’s prediction (71). In this framework, each feature is treated as a “player” in a game, and the model prediction is the “payout” distributed among them based on their marginal contributions. For example, in the study by Novielli et al. (73), SHAP was used to analyze the drivers of soil respiration sensitivity (*Q*_10_). The results revealed that glucose-induced soil respiration and the proportion of bacterial taxa positively associated with *Q*_10_ were among the most influential predictors. SHAP quantified the contribution of these features by measuring how the model’s output changed with and without each feature, providing insights aligned with established ecological understanding, such as the role of microbial metabolism in carbon cycling. This demonstrates how SHAP can help uncover key biological drivers in complex environmental systems. While SHAP as a technique is extremely robust, due to its game-theoretic foundations, its explanations still rely on the model’s behavior under perturbations—a vulnerability shared with LIME that adversarial attacks can exploit. Slack et al. (74) demonstrated how classifiers can be deliberately engineered to deceive *post hoc* explanation methods, with LIME proving particularly susceptible due to its local approximation approach compared to SHAP’s. These findings reveal fundamental vulnerabilities in current perturbation-based explanation methods, demanding new research directions in adversarially robust explainability that can maintain interpretability while withstanding manipulation attempts.

**Fig 1:**
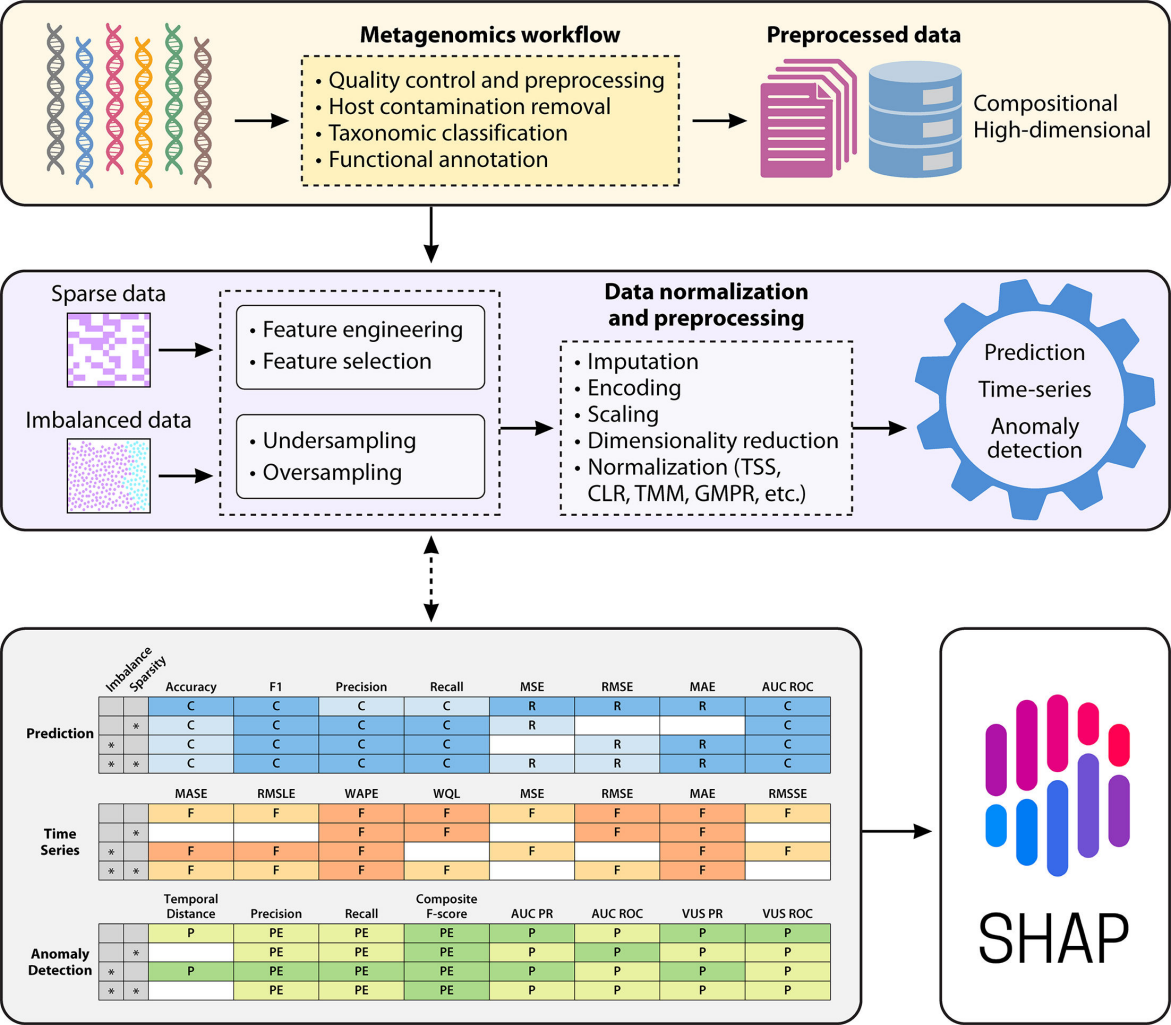
Overview of the ML workflow for metagenomic data analysis. The sequencing data are first processed through the metagenomics workflow to generate features that are inherently compositional and high-dimensional. Depending on data characteristics such as sparsity or class imbalance, appropriate preprocessing steps are applied to homogenize and prepare the data for ML. Based on the study objectives (prediction, time-series, or anomaly detection), an appropriate ML model is chosen and evaluated using the most suitable scoring metric. To obtain biological insights, interpretability techniques such as SHAP are employed.

Given these limitations, it is essential to quantitatively evaluate ML models. In microbiome research, assessing models using appropriate performance metrics is a standard best practice. For readers interested in how to evaluate ML models in microbiome applications, we provide a comprehensive overview of relevant metrics and their appropriate use cases in Note S1, along with a task-metric scoring matrix (Table S1) and a glossary of terms (Table S2). In Fig. 1, we present a general workflow for implementing ML for metagenomic data.

Most critically, these challenges underscore that XAI aims to explain the inner logic and choice-making of an ML model, shedding light on how a model came to its prediction. Explanations derived in this manner do not have to mirror the underlying biological processes. As a result, there are likely to be differences between outputs from models and biological ground truth. Hence, domain expert validation is crucial to establish the reliability and biological significance of the conclusions inferred from these models.

## COMPARING AND COMBINING ML AND MECHANISTIC MODELS IN METAGENOMICS

Before the rise of ML, microbiome research relied heavily on mechanistic models such as Boolean models, ordinary differential equations (ODEs), and constraint-based models to describe microbial dynamics and interactions. Although this review has focused on ML for the analysis of metagenomic data, mechanistic models continue to play an important complementary role (75, 76). Both come with their strengths and limitations, and the decision to use one over the other should be based on the nature and quality of the data set, as well as the objectives of the study. While ML excels at identifying non-linear and complex patterns in large-scale, high-dimensional, and time-series data sets, it often fails to infer causality. Mechanistic models, on the other hand, are rooted in hypothesis generation and causal reasoning, aiming to capture the underlying processes driving microbial community dynamics (77, 78). For instance, while an ML model may distinguish between healthy and diseased microbiome states, a mechanistic model can elucidate how changes in nutrient concentrations lead to such shifts. A recent study by Kuppa Baskaran et al. (79) applied this approach by constructing metagenome-based metabolic models to predict metabolic exchanges and horizontal gene transfer events in the deep-sea hydrothermal vents’ microbiome, offering insights into archaeal-bacterial interactions that underpin community structure. However, these models can be limited by their underlying assumptions and may not readily scale to complex data sets. Their development also demands domain-specific expertise and significant manual effort in curation and analysis. This has spurred interest in causal ML, which aims to extract causal relationships directly from observational data with minimal assumptions.

Causal ML offers a middle ground by bridging the correlation-causation gap. It extracts associations and potential causal relationships from data, adding to the interpretability of the results. DoWhy (80) is one framework that explicitly models causal assumptions and validates them, unlike traditional ML models. The advantage is that it reduces false positives due to spurious correlations and allows for designing mechanistic follow-ups of the results.

Another clever way to deploy ML is as a hybrid strategy with mechanistic or statistical models. A compelling example of this hybrid philosophy is mbtransfer, a method that blends ML with principles from control theory to study how interventions reshape microbial communities over time. Unlike conventional ML, which treats causality as an afterthought, mbtransfer embeds temporal reasoning into its framework: it uses transfer functions—a concept adapted from engineering to model delayed effects of perturbations (e.g., dietary changes or birth events)—and simulates counterfactual scenarios (e.g., “What if the intervention had not occurred?”). These simulations, combined with mirror statistics (a statistical method that controls false discoveries by comparing results across data splits), allow researchers to identify taxa most sensitive to interventions. While not a fully mechanistic model, mbtransfer bridges ML’s scalability with causal ML’s ambition, inferring when and how interventions matter (45). The resulting hypotheses can then be tested mechanistically (e.g., using metabolic models or generalized Lotka-Volterra equations), creating a feedback loop where ML-driven discoveries inform biological validation. This mirrors frameworks like DoWhy but directly addresses microbiome-specific challenges, such as sparse time-series data and phylogenetic dependencies (80).

It is important to recognize that neither ML nor mechanistic models can compensate for the low-quality data or a flawed experimental design. An adequate sample size per experimental group is essential for both approaches. Both approaches demand sufficient sample sizes to yield reliable results. ML methods are particularly vulnerable to overfitting when applied to small data sets. On the other hand, mechanistic models require adequate observations to constrain parameters and validate assumptions. This brings us to statistical significance, often expressed through *P-*values, a concept developed to help researchers communicate confidence in their results. Unfortunately, *P*-values have been widely misused and even abused, intentionally or otherwise. Studies in the metagenomics space are often underpowered and increasingly vulnerable to *P*-hacking, where researchers test multiple hypotheses to find one that appears significant, despite the volume of data generated (81). The low sample size only worsens this, increasing the risk of false positives or negatives. Kers and Saccenti’s study is one of the few studies that explicitly discuss how small sample sizes can skew alpha-diversity metrics and emphasize the need for statistical planning before the experiment begins. As a general guideline, a minimum of 25 samples per category is recommended for any analysis, whether the approach is mechanistic or ML-based (82).

Ultimately, it is not a question of choosing one approach over the other, but how we could strategically combine them to extract predictive power and mechanistic insights from microbiome data. Such synergies between ML and mechanistic modeling have been well explored in drug discovery and related domains (83), and there is much opportunity to carry over such learnings to metagenomic data analysis.

## FUTURE PERSPECTIVES

The idea that genes are the codes that shape the trajectories of our lives is only half the truth. Genes do not function autonomously; instead, they are akin to a database that the biological system accesses and interprets contextually, rather than a program that executes independently. Metagenomics thus gives us a very static view of microbial DNA. To truly understand function, context, and dynamics, we must move beyond the DNA picture to include metatranscriptomics (RNA expression), metaproteomics (proteins in action), and metabolomics (the results of all activity). These layers reveal what microbes are actively doing in a given environment. To meet the complexity of such biological questions, computational tools must evolve accordingly. MMAPLE is one such tool, a multimodal, multi-omics framework that combines various data types and employs a meta-learning approach, promising results in extracting meaningful insights from complex microbiome data sets (7). Hence, new ML models tailored for multi-omics integration and analysis represent the next frontier in decoding microbial function, context, and dynamics.

However, integrating these diverse data sets is not straightforward. While multi-omics integration is conceptually promising, it is technically very challenging. Aligning heterogeneous data with different scales, missing values, noise characteristics, and batch effects poses a substantial barrier to seamless integration, demanding innovative normalization or imputation strategies. Furthermore, as multi-omics, single-cell, temporally and spatially resolved data sets grow, computational efficiency can become a limiting factor. Training large-scale models—especially with DL frameworks—requires significant compute, memory, time, and energy resources, which may be inaccessible to smaller research groups.

In parallel to traditional sequence-based metagenomics, recent efforts are beginning to explore the untapped potential of raw signal data produced by sequencing technologies. For instance, Urel et al*.* (84) developed a DL framework that infers microbial viability directly from raw nanopore electrical signals, bypassing the need for read-level taxonomic assignment. These signal-based ML models offer new possibilities for identifying functional states of microbes (e.g., live vs. dead) and present unique preprocessing challenges such as denoising, segmentation, and feature extraction from unstructured time-series data. As sensor-based metagenomics grows, developing robust signal processing and evaluation metrics tailored to such data will be essential.

Another emerging frontier in artificial intelligence (AI) is the development of agentic AI systems. These hybrid frameworks combine the structured logic and deterministic nature of software engineering with the adaptability of AI. Recent innovations such as AgentClinic (85), Agent Laboratory (86), and the AI co-scientist (87) exemplify this shift toward systems that not only analyze data but actively assist in experimental design, hypothesis generation, and interpretation. Such systems could play a vital role in microbiome research and help explore interventions or simulate ecological shifts *in silico*.

Alongside agentic AI, generative models and LLMs are now powering synthetic data for microbiome studies. Tools like Evo 2, for example, can infer and “fill in” low‑coverage regions of MAGs from sparse reads (68), producing biologically plausible genomes. These reconstructed sequences can then be used to test how particular mutations or environmental shifts affect metabolic outputs, giving us an *in silico* window into evolutionary dynamics. Of course, each synthetic data set must be benchmarked against real measurements to guard against bias and ensure the models we build remain grounded in biology.

Despite the rapid rise of ML in metagenomics, many challenges remain to be addressed. First, there is a lack of standardized benchmarks for evaluating models across diverse microbiome data sets—sample sizes, sequencing platforms, annotation depths, and environmental contexts all vary wildly, making comparisons across studies difficult. Second, reproducibility suffers when studies omit detailed documentation or fail to share containerized workflows. We urgently need open‑source tools, shareable pipelines, and strict adherence to findable, accessible, interoperable, reusable, reproducible (FAIReR) data principles (88). Finally, public data are heavily skewed toward European and North American cohorts—the South American MicroBiome Archive (saMBA) preprint reports that over 70% of sequenced human microbiomes come from these regions (89). Such geographic and population biases threaten the global generalizability of our ML models.

Overall, ML models and emerging advances in AI will have a telling impact on microbiome data analysis. The next frontier lies in hybrid approaches that marry ML with mechanistic models and statistical frameworks. As cartographers of the microbial world, we must summon every kind of map: from multi-omics integration that layers genomic, proteomic, and metabolic data, to algorithmic compasses that blend deep learning with ecological theory. Only by wielding this full spectrum of tools can we truly navigate the boundless complexity of these invisible ecosystems that quietly govern life on Earth.

## SUPPLEMENTAL MATERIAL

Supplemental Material msystems.01642-24-s0001.pdf Scoring matrix for evaluating machine learning models.

ASM does not own the copyrights to Supplemental Material that may be linked to, or accessed through, an article. The authors have granted ASM a non-exclusive, world-wide license to publish the Supplemental Material files. Please contact the corresponding author directly for reuse.
