# Supplementary material for: Harnessing machine learning for metagenomic data analysis: trends and applications
Source: mSystems. 2025 Oct 7;10(11):e01642-24. doi: 10.1128/msystems.01642-24 (PMC12625703; doi:10.1128/msystems.01642-24)
Supplement: Supplemental Material — Scoring matrix for evaluating machine learning models. [file msystems.01642-24-s0001.pdf]

Harnessing Machine Learning for Metagenomic Analysis: Trends and Applications

Supplementary Information

Shradha Sharma[1,2,3], Hari Priya Narahari[3,4], and Karthik Raman*[3]

[1]Bhupat and Jyoti Mehta School of Biosciences, Indian Institute of Technology Madras, Chennai 600036, Tamil Nadu, India
[2]The Centre for Integrative Biology and Systems medicinE (IBSE), Indian Institute of Technology Madras, Chennai 600036, Tamil Nadu, India
[3]Department of Data Science and AI, Wadhwani School of Data Science and AI, Indian Institute of Technology Madras, Chennai 600036, Tamil Nadu, India
[4]Department of Computer Science and Engineering, Shiv Nadar University, Chennai, Chennai 603110, Tamil Nadu, India

Supplementary Note S1: Evaluating Machine Learning Models

Implementing machine learning in a metagenomics dataset is not a one-step process. It is a sequential process that involves preprocessing of the data to deal with sparsity, compositional, and high-dimensionality of the sequencing data. Once the data are prepared, they are fed into the appropriate machine learning model [1]. It should be noted that, while certain combinations of transformation methods and machine learning models may perform slightly better, the overall accuracy often depends more on the characteristics of the data itself (e.g., sequencing depth, sample size) rather than the transformation method. However, the choice of transformation can influence which taxa are identified as necessary by the models, even if the accuracy remains similar. Since no single algorithm consistently outperforms others in metagenomics data, as supported by the No Free Lunch Theorem, careful selection of the transformation method and model combination is warranted based on the specific data and outcome goals [2, 3].

A striking example of how preprocessing and modeling choices can lead to misleading conclusions is provided by the reanalysis of cancer microbiome studies by Gihawi et al. [4], where near-perfect classification accuracy was reported by Poore et. al [5] but later found to arise from misidentified human reads and flawed preprocessing. This underscores the need for rigorous and transparent evaluation practices.

To ensure that machine learning workflows in microbiome research are reliable, unbiased, and generalizable, the results must be rigorously evaluated. Over time, various evaluation metrics have been developed to assess how well a model performs, whether it is doing well or poorly, and to enable meaningful comparisons between models trained on the same dataset.

While building ML models, it is natural to gravitate towards standard metrics like accuracy and F1 score, which may not fully capture the pros and cons of the model. Therefore, based on the kind of data and applications, we must choose an appropriate metric (not necessarily a single metric) to evaluate the models' performance. For instance, an ML model built on a highly imbalanced vaginal microbiome dataset to predict term or pre-term birth, where pre-term samples are extremely low-might achieve a result of 90% when evaluated on accuracy. However, in reality, this accuracy might fail to capture the pre-term birth conditions at all. Hence, a combination of metrics like AUC ROC, F1-Score, Recall, and balanced accuracy would be a better choice to avoid bias and capture the model's performance than a simple accuracy evaluation [6].

Choosing the right metric is highly dependent on the nature of the dataset and the modeling objective. Given the wide range of options available, we have created a table to help guide the selection

*Corresponding author: `kraman@dsai.iitm.ac.in`

of appropriate metrics based on the type of task and the data involved. The papers [1, 7] provide an overview of commonly used metrics for classification and regression, along with their applications and pitfalls in microbiome studies.

Following the pipeline to use ML for microbiome analysis (Table 1), it is a good practice to use evaluation metrics as per the data and application. We propose a scoring metric that can be referred to (Figure S1).

The table is organized into three main sections: Prediction, Time Series, and Anomaly Detection. Each section lists commonly used metrics, with further subcategories based on the type of task.

- For Prediction, the metrics are divided into Classification (for categorical outcomes) and Regression (for continuous values).
- The Time Series section focuses on metrics used for Forecasting tasks.
- The Anomaly Detection section is split into Point-based and Event-based metrics. Point-based metrics evaluate individual data points that deviate from the norm, while event-based metrics assess unusual patterns across sequences of events.

A closer examination of the table reveals that certain metrics can be applied across various types of data. For predictive modeling, the F1 score and AUC-ROC provide reliable results regardless of the data type. When it comes to time series data models, WAPE (Weighted Absolute Percentage Error) and MAE (Mean Absolute Error) are the preferred metrics. Additionally, the composite F-score offers a concise summary of an anomaly detection system.

	Imbalance	Sparsity	Accuracy	F1	Precision	Recall	MSE	RMSE	MAE	AUC ROC
Prediction			C	C	C	C	R	R	R	C
		*	C	C	C	C	R			C
	*		C	C	C	C		R	R	C
	*	*	C	C	C	C	R	R	R	C

	Imbalance	Sparsity	MASE	RMSLE	WAPE	WQL	MSE	RMSE	MAE	RMSSE
Time Series			F	F	F	F	F	F	F	F
		*			F	F		F	F	
	*		F	F	F		F		F	F
	*	*	F	F	F	F		F	F	

	Imbalance	Sparsity	Temporal Distance	Precision	Recall	Composite F-score	AUC PR	AUC ROC	VUS PR	VUS ROC
Anomaly Detection			P	PE	PE	PE	P	P	P	P
		*		PE	PE	PE	P	P	P	P
	*		P	PE	PE	PE	P	P	P	P
	*	*		PE	PE	PE	P	P	P	P

Table S1: **Scoring matrix for machine learning algorithms used in microbiome analysis.** The matrix maps commonly used evaluation metrics to different ML task types: **Prediction**, **Time Series**, and **Anomaly Detection**. The **Prediction** section is further divided into *Classification* C (for categorical outcomes) and *Regression* R (for continuous outcomes). The **Time Series** section includes metrics used in *Forecasting* F tasks. The **Anomaly Detection** section is split into *Point-based* P and *Event-based* E metrics, with some applicable to both PE. Point-based metrics evaluate individual anomalies, while event-based metrics assess unusual patterns over sequences. Task types are annotated as: C (Classification), R (Regression), F (Forecasting), P (Point-based), E (Event-based), and PE (both). Darker cells represent **recommended or particularly informative metrics** for each task type, while lighter cells indicate metrics that are useful in specific scenarios.

Term	Definition
Prediction	The model's ability to estimate biological outcomes such as health status, host phenotype, or microbial composition from input features (e.g., taxa).
Time series	Analysis of data collected at successive time intervals to capture temporal dynamics. Such data in microbiome studies can reveal microbial succession patterns, stability, or the response to environmental changes over time.
Anomaly detection	Identification of outliers from a sample. In microbiome studies, anomaly detection can help identify microbial profiles that deviate from expected behavior, potentially indicating contamination or a diseased state.
Imbalance	A characteristic of a dataset wherein one category contains significantly more samples than another. This affects the model's performance in microbiome studies, especially where rare conditions are underrepresented.
Sparsity	Presence of a high proportion of missing values or zeros in a dataset. In microbiome studies, it arises due to low-abundance or undetected taxa across samples [1].
Accuracy	Proportion of correct predictions made by the model out of all predictions. For example, classification studies use accuracy to distinguish between healthy and diseased states based on microbial data [8].
F1 score	A performance metric that calculates the harmonic mean of precision and recall value. It is useful in microbiome studies to deal with class imbalance, as it balances the trade-off between false positives and false negatives [8].
Composite F-score	An aggregated F1 score is used when evaluating multiple labels or classes [9]. For example, in a study that aimed to classify samples into healthy, IBD (Inflammatory Bowel Disease), and IBS (Irritable Bowel Syndrome), a composite F1 score would summarize the model's performance across the three conditions. This allows us to assess the overall performance of the classifier rather than just one condition.
Precision	Proportion of true positive predictions out of all positive predictions made by a model. It indicates how many of the samples predicted to belong to a certain class are correct-that is, how accurate the positive predictions are. For example, in classifying healthy vs diseased samples, precision reflects how often a model is correct when predicting a diseased sample [8].
Recall	Proportion of true positive predictions from all actual positives in a dataset. It indicates how well a model has identified all relevant samples in a given class. For example, in a task classifying diseased vs. healthy samples, recall measures how many of the actual diseased samples the model correctly detected—that is, how well it avoids missing positives. [1].
RMSE	A Root Mean Squared Error is the average magnitude of prediction errors in regression tasks. It is calculated as the square root of the average of squared differences between predicted and actual values [8].
MAE	A Mean Absolute Error is the average absolute difference between predicted and actual values in regression tasks. It is less sensitive to outliers [8].
MASE	Mean Absolute Scaled Error is a metric for evaluating prediction accuracy, particularly in time-series forecasting. It is calculated as the mean absolute error (MAE) of the model, scaled by the MAE of a naïve baseline forecast (typically the previous time step). This scaling makes MASE scale-independent and allows for comparison across different datasets. In microbiome studies, it can be used to assess the accuracy of temporal predictions such as changes in microbial abundances over time [8].

Term	Definition
RMSLE	A Root Mean Squared Logarithmic Error is a regression metric that measures the squared logarithmic differences between predicted and actual values, then takes the square root. It penalizes under-predictions more than over-predictions and is less sensitive to large errors when values are high. RMSLE is useful when the target variable spans several orders of magnitude [10].
WAPE	A Weighted Absolute Percentage Error is a prediction accuracy metric computed as the sum of absolute errors divided by the sum of actual values, often expressed as a percentage. WAPE adjusts for the scale of the data, making it interpretable across datasets. In microbiome studies, it is useful for evaluating models that predict relative abundances or other compositional outputs [11].
WQL	A Weighted Quantile Loss is a function used to evaluate quantile regression models by penalizing over- and under-predictions differently based on a specific quantile. The "weighted" aspect allows assigning different importance to different prediction intervals or data segments [12].
MSE	A Mean Squared Error is a metric that evaluates the performance of predictive models by measuring the average squared difference between predicted and actual values. It penalizes larger errors more than smaller ones, making it sensitive to outliers [8].
RMSSE	A Root Mean Squared Scaled Error is an extension of RMSE, calculated by dividing the RMSE by the RMSE of a naive forecast, typically assuming that the next value will be equal to the previous one. It is a scale-free measure of forecast accuracy, enabling easier comparison of performance across different datasets or models. [10].
Temporal Distance	The time interval between two data points in time-series data.
AUC ROC	The Area under the ROC (Receiver Operating Characteristic) curve is a metric that measures a model's ability to distinguish between classes across all classification thresholds. The ROC curve plots the true positive rate against the false positive rate. A higher AUC indicates better discrimination between classification groups [9].
AUC PR	The Area under the precision-recall curve. It is a metric that measures how well a classifier balances precision and recall across different thresholds. Unlike AUC ROC, it focuses only on the positive class, making it preferable when the dataset is imbalanced. It is beneficial for evaluating how effectively a model has identified the minority class [9].
VUS PR	The Volume under the precision-recall surface is a metric employed to evaluate multiclass classification performance. It is helpful in capturing the balance between precision and recall across all classes, making it valuable in imbalanced datasets [9].
VUS ROC	The Volume under the receiver-operating curve is an extension of AUC ROC as it is used for multiclass problems. It evaluates how well a classifier ranks true classes higher than false ones across all possible class combinations. The difference between the composite F1 score and VUS ROC can be tricky to understand. To simplify: composite F1 scores evaluate how accurately the predictions match the true labels, while VUS ROC evaluates how confidently the model ranks the true class over the others [9].

Table S2: **Glossary for the terms used in the scoring matrix.**

References

[1] Corinne Walsh, Elías Stallard-Olivera, and Noah Fierer. Nine (not so simple) steps: a practical guide to using machine learning in microbial ecology. *MBio*, 15(2):e02050–23, 2024.

[2] Zuzanna Karwowska, Oliver Aasmets, Estonian Biobank research team Metspalu Mait Metspalu Andres Milani Lili Esko Tõnu, Tomasz Kosciolek, and Elin Org. Effects of data transformation and model selection on feature importance in microbiome classification data. *Microbiome*, 13(1):2, 2025.

[3] David H Wolpert and William G Macready. No free lunch theorems for optimization. *IEEE transactions on evolutionary computation*, 1(1):67–82, 1997.

[4] Abraham Gihawi, Yuchen Ge, Jennifer Lu, Daniela Puiu, Amanda Xu, Colin S Cooper, Daniel S Brewer, Mihaela Pertea, and Steven L Salzberg. Major data analysis errors invalidate cancer microbiome findings. *MBio*, 14(5):e01607–23, 2023.

[5] Gregory D Poore, Evguenia Kopylova, Qiyun Zhu, Carolina Carpenter, Serena Fraraccio, Stephen Wandro, Tomasz Kosciolek, Stefan Janssen, Jessica Metcalf, Se Jin Song, et al. Retracted article: Microbiome analyses of blood and tissues suggest cancer diagnostic approach. *Nature*, 579(7800):567–574, 2020.

[6] Jonathan L Golob, Tomiko T Oskotsky, Alice S Tang, Alennie Roldan, Verena Chung, Connie WY Ha, Ronald J Wong, Kaitlin J Flynn, Antonio Parraga-Leo, Camilla Wibrand, et al. Microbiome preterm birth dream challenge: Crowdsourcing machine learning approaches to advance preterm birth research. *Cell Reports Medicine*, 5(1), 2024.

[7] Catriona Miller, Theo Portlock, Denis M Nyaga, and Justin M O'Sullivan. A review of model evaluation metrics for machine learning in genetics and genomics. *Frontiers in Bioinformatics*, 4:1457619, 2024.

[8] Aurélien Géron. *Hands-on machine learning with Scikit-Learn, Keras, and TensorFlow: Concepts, tools, and techniques to build intelligent systems.* " O'Reilly Media, Inc.", 2022.

[9] Sondre Sørbø and Massimiliano Ruocco. Navigating the metric maze: A taxonomy of evaluation metrics for anomaly detection in time series. *Data Mining and Knowledge Discovery*, 38(3):1027–1068, 2024.

[10] Rob J Hyndman and Anne B Koehler. Another look at measures of forecast accuracy. *International journal of forecasting*, 22(4):679–688, 2006.

[11] Hansika Hewamalage, Klaus Ackermann, and Christoph Bergmeir. Forecast evaluation for data scientists: common pitfalls and best practices. *Data Mining and Knowledge Discovery*, 37(2):788–832, 2023.

[12] Tilmann Gneiting and Matthias Katzfuss. Probabilistic forecasting. *Annual Review of Statistics and Its Application*, 1(1):125–151, 2014.
